# Case Report: Severe Neonatal Course in Paternally Derived Familial Hypocalciuric Hypercalcemia

**DOI:** 10.3389/fendo.2021.700612

**Published:** 2021-10-01

**Authors:** Jakob Höppner, Sabrina Lais, Claudia Roll, Andreas Wegener-Panzer, Dagmar Wieczorek, Wolfgang Högler, Corinna Grasemann

**Affiliations:** ^1^ Department of Pediatrics, St Josef-Hospital Bochum, Ruhr-University Bochum, Bochum, Germany; ^2^ Department of Neonatology, Pediatric Intensive Care and Sleep Medicine, Vestische Kinder- und Jugendklinik Datteln, University Witten/Herdecke, Datteln, Germany; ^3^ Department of Radiology, Vestische Kinder- und Jugendklinik Datteln, University Witten/Herdecke, Datteln, Germany; ^4^ Institute of Human Genetics, Medical Faculty and University Hospital Düsseldorf, Heinrich-Heine-University Düsseldorf, Düsseldorf, Germany; ^5^ Department of Paediatrics and Adolescent Medicine, Johannes Kepler University Linz, Linz, Austria

**Keywords:** familial hypocalciuric hypercalcemia, neonatal hyperparathyroidism, pregnancy, management, calcium sensing receptor (CaSR), FHH

## Abstract

Familial hypocalciuric hypercalcemia (FHH, [OMIM #145980]) is recognized as a benign endocrine condition affecting PTH and calcium levels due to heterozygous inactivating mutations in the calcium sensing receptor (*CaSR*). The condition is often un- or misdiagnosed but may have a prevalence as high as 74 in 100.000. Here, the neonatal courses of two brothers with paternally inherited FHH (*CaSR* c.554G>A; p.(Arg185Gln)) are described. The older brother was born preterm at 25 weeks gestation with hypercalcemia and hyperparathyroidism. The younger brother, born full-term, had severe hyperparathyroidism, muscular hypotonia, thrombocytopenia, failure to thrive and multiple metaphyseal fractures. Treatment with cinacalcet was initiated, which resulted in subsequent reduction of PTH levels and prompt clinical improvement. While it is known that homozygous mutations in *CaSR* may lead to life-threatening forms of neonatal severe hyperparathyroidism (NSHPT), few reports have described a severe clinical course in neonates with FHH due to heterozygous mutations. However, based on the pathophysiological framework, in *de novo* or paternally transmitted FHH the differing calcium needs of mother and fetus can be expected to induce fetal hyperparathyroidism and may result in severe perinatal complications as described in this report. In summary, FHH is a mostly benign condition, but transient neonatal hyperparathyroidism may occur in affected neonates if the mutation is paternally inherited. If severe, the condition can be treated successfully with cinacalcet. Patients with FHH should be informed about the risk of neonatal disease manifestation in order to monitor pregnancies and neonates.

## Introduction

Loss-of-function mutations in the calcium-sensing and signaling pathway cause a spectrum of calcium-hyposensitivity-disorders with elevated PTH secretion (1). The resulting disorders can be summarized by inappropriately high PTH concentration despite elevated serum calcium levels.

Familial hypocalciuric hypercalcemia (FHH) comprises a genetically heterogenic group (2): FHH1 [OMIM #145980] is caused by heterozygous *inactivating* mutations in the calcium-sensing receptor (*CaSR*) gene (3). FHH2 and 3 are caused by mutations in genes encoding for proteins involved in calcium signal transduction (4–6). The CaSR is a member of the subfamily of G protein-coupled transmembrane receptors and is expressed in parathyroid chief cells and the renal tubulus (7). FHH typically presents with the biochemical triad: life-long, non-symptomatic, non-progressive hypercalcemia, normal or slightly elevated serum PTH levels and hypocalciuria (8). Thus, FHH is thought to be a mostly benign condition, with no definite association to adverse outcomes (8). Accordingly, the vast majority of patients with FHH do not require medical or surgical treatment (2, 9) and counseling of affected individuals aims to avoid future misdiagnosis and unnecessary parathyroidectomies (9, 10).

In contrast to the clinically benign course of FHH1, neonatal severe primary hyperparathyroidism (NSHPT) [OMIM #239200] is a severe rare disease associated with a high mortality and is usually caused by homozygous inactivating mutations in the CaSR gene (8, 11). Infants with NSHPT develop severe and symptomatic hypercalcemia with muscular hypotonia, respiratory distress, fractures, intestinal dysmotility, and failure to thrive in the early days of life (12–14). Milder phenotypes are summarized as neonatal hyperparathyroidism (NHPT) and refer to infants with elevated serum PTH levels and resulting bone disease, but only modest hypercalcemia, usually based on heterozygous inactivating mutations of *CaSR* (15).

Here, we present the disparate neonatal courses of two siblings with a paternally inherited FHH.

## Case Presentation

Patient 1 was born in 2017 and is the first child of unrelated Caucasian parents. He was born by cesarean section at 25 weeks after premature rupture of membranes with gestational age-appropriate weight and height.

**Figure 1:**
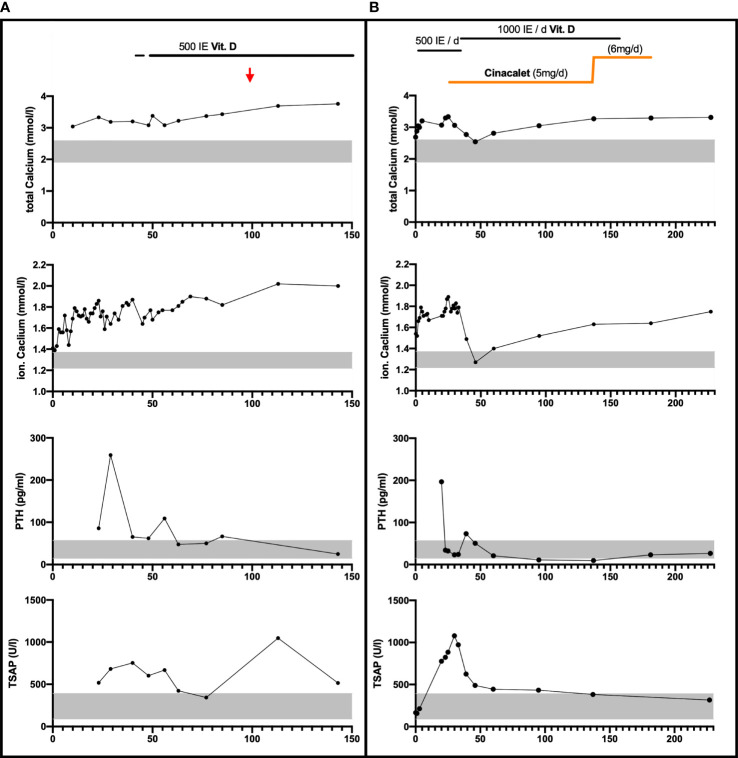
Laboratory parameters and calcium-affecting treatments over the first 150 respectively 240 days in Patient 1 Panel **(A)** and Patient 2 Panel **(B)**. Vitamin D3 was provided orally/once daily. Cinalcacet was started in patient 2 on day 26 of life at 20 mg/m² body surface (5mg) daily and increased on day 137 of life based on increased body weight and elevated calcium levels (to 6mg). Age-appropriate normative range is indicated by the grey bar for calcium, PTH and total serum alkaline phosphate (TSAP) levels; Red arrow: estimated date of delivery.

At birth, his serum calcium was 3.04 mmol/l (n: 1.90 – 2.60) and ionized calcium 1.41 mmol/l (n: 1.22 – 1.37). At day 23 of life elevated PTH (85.9 pg/ml [n: 14.9 – 56.9]) and total serum alkaline phosphatase (TSAP) (518 U/l [n: 89 – 390]) were detected while urinary calcium excretion was low (**Figure 1A**). Phosphate levels were within the normal range. He required tube-feeding with breastmilk starting day 1 of life. During the first 2 weeks, he received partial parenteral nutrition with lipids containing vitamin D3. Due to hypercalcemia routine oral vitamin D and calcium supplementation were withheld until day 42 of life.

Based on a suspected diagnosis of FHH the routine supplementation with 500 IE Vitamin D3 daily was initiated from day 42 and a calcium supplementation of 128 mg/kg BW/d (from day 48 to age 3 months) was started as appropriate for preterm infants. Calcium levels stabilized around 3.42 mmol/l and PTH remained only slightly elevated (**Figure 1A**).

At discharge at a postmenstrual age of 38 weeks renal ultrasound showed minimal nephrocalcinosis.

Molecular genetic analysis revealed the same heterozygous variant in *CaSR* NM_000388.4:c.554G>A;p.Arg185Glu;[GRCh37/hg19:chr3:g.121980436], a class V variant, that was later proven in his father. The father has been asymptomatic so far and no further diagnostic workup has been performed. To our knowledge, no other family members are affected.

Bayley Scales of Infant Development-II showed normal development at 24 months corrected age (Mental Development Index 112, Psychomotor Developmental Index 96). The boy is a currently a healthy 4-year-old with a stable serum calcium of around 3.6 mmol/l. No nephrocalcinosis is present on ultrasound.

Patient 2 is the family’s second child. He was born by vaginal delivery at a gestational age of 41 weeks with normal size and Apgar scores.

At 31 and 37 weeks of gestation bowing of the right proximal femur was detected by ultrasound. Postnatally, clinical examination revealed radial deviation of both wrists.

Laboratory examination on day 1 showed thrombocytopenia (15.000/μl [n: 355.000 – 666.000/μl]) and hyperbilirubinemia (9.5 mg/dl [n: 0 – 7.0 mg/dl]). The boy received two platelet transfusions and phototherapy. Elevated serum calcium of 2.69 mmol/l and an ionized calcium with 1.54 mmol/l was present. PTH was elevated at 196.5 pg/ml, while serum phosphate and TSAP were normal. Urinary calcium was undetectable when first measured on day 21 of life (**Figure 1B**). According to national guidelines routine vitamin D3 supplementation with 500 IE/d was started on day 5.

**Figure 2:**
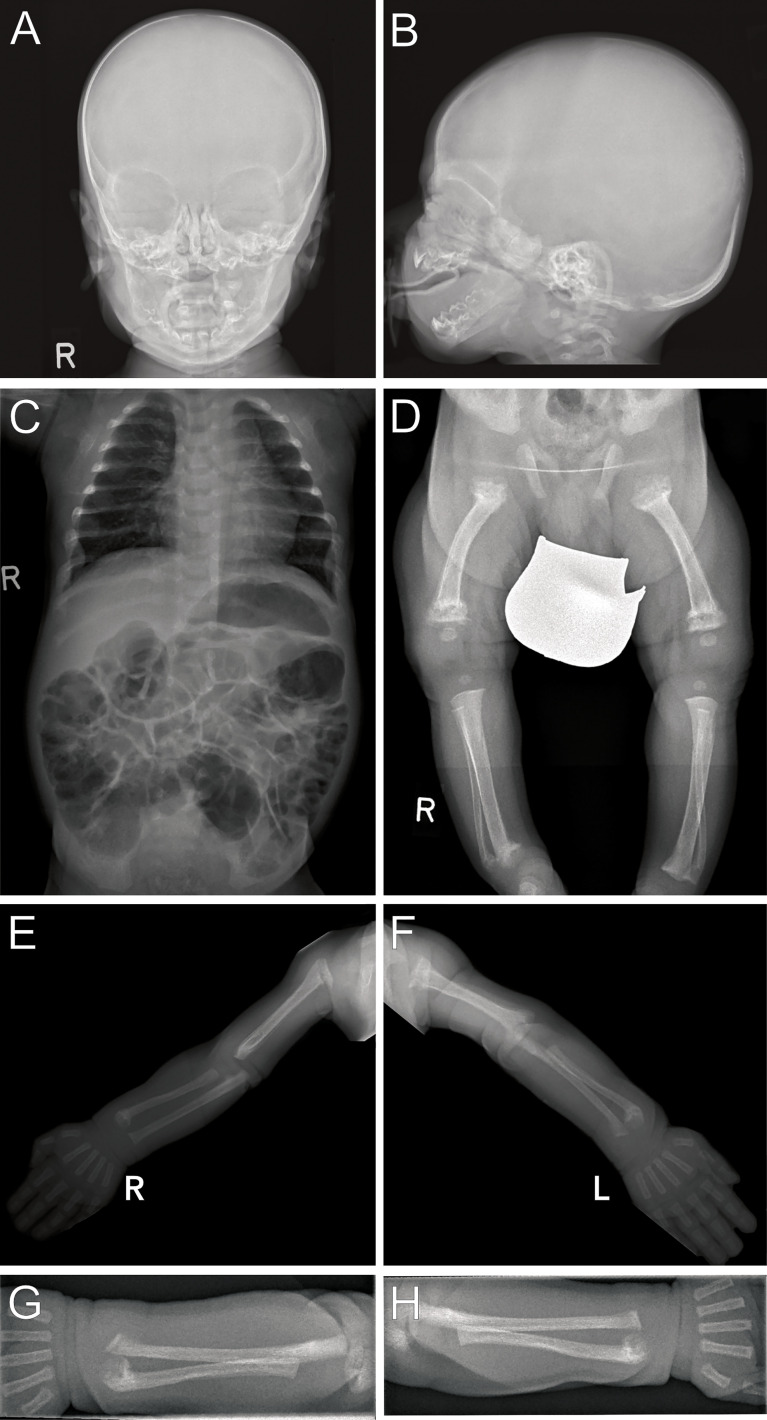
Radiographs (Patient 2) at 3 weeks of life: Images of the skull **(A, B)** show blurred borders at the cortical/medullar bones and wide sutures with occipital intrasutural bones (Wormian bones). Poor mineralization is present in the skeleton as seen on the thoracoabdominal radiograph **(C)** arms and legs. Multiple metaphyseal fractures are present in both arms, with malalignments of the radius and ulna **(E–H)**. Distal fractures of the right tibia and fibula are present **(D)**, with internally rotated position and, to a lesser extent, likewise on the left. Fractures on the left side are close to consolidation, indicating intra uterine onset of fractures.

Radiographs revealed multiple metaphyseal fractures of the long bones as well as multiple fractures at different stages of healing. Skeletal survey showed a generalized decrease in bone density with poor mineralization of the entriegeln skeleton, and at the skull wide sutures and a wormian bone was noted. (**Figure 2**).

The boy showed muscular hypotonia, limited movement of extremities, mispositioning of both wrists and the left proximal humerus with signs of discomfort.

The familial diagnosis of FHH was assumed and later confirmed. Patient 2 carries the same mutation in *CaSR* as his older brother and his father: NM_000388.4:c.554G>A;p.Arg185Glu;[GRCh37/hg19:chr3:g.121980436].

Breastfeeding was attempted, but proved difficult due to muscular hypotonia.

The patient was started on 20 mg/m² body surface/day of Cinacalcet orally given as 2.5mg twice daily on day 26. Subsequently, PTH levels decreased and urinary calcium excretion was detectable. The patient’s overall clinical condition gradually improved. (**Figure 1B**).

Breastmilk was provided *via* bottle feeds and routine vitamin D supplementation was increased to 1000 IE per day during the 4^th^ week of life.

Maternal blood test (on day 23 after delivery) showed normal levels of 25OH vitamin D at 22.0 ng/ml [n: 20 - 46] calcium (2.36 mmol/l [n: 2.20 – 2.70]), phosphate (1.31 mmol/l [n: 1.10 – 2.00]) and PTH at 18.4 pg/ml [n: 15 – 65].

On follow-up, the boy presented bi-weekly from 6 weeks of age until present (age 6 months). Muscular hypotonia gradually improved, as did the deformities on the left proximal humerus and the wrists. Cinacalcet was discontinued at 6 months of age and the vitamin D supplementation was reduced to 500 IE/d, to prevent oversubstitution. Serum calcium currently is 3.20 mmol/l and PTH is 26.7 pg/ml. There is no evidence for nephrocalcinosis (**Figure 1B**).

## Discussion

Whilst FHH1 [OMIM #145980] is thought to be a benign, typically asymptomatic condition (8), transient neonatal hyperparathyroidism later reverting to FHH has been reported, even in patients with heterozygous *CaSR* mutations, including the c.554G>A, p.Arg185Gln; R185Q variant present in the family of this report (13, 16–21). This mutation is known to result in defective receptor signaling (13, 22, 23), while the ligand binding is unaffected allowing for potential treatment with calcimimetic drugs (12, 24).

The development of neonatal hyperparathyroidism in FHH is likely modified most significantly by the parental origin of the *CaSR* mutation. In pregnancies affected by *de novo* or paternally derived mutations, the calcium setpoints of mother and fetus differ and result in conflicting regulation of calcium levels (13, 23). In these pregnancies the normocalcemic maternal environment is perceived as hypocalcemic by the fetus and thus induces fetal hyperparathyroidism to support increased fetal calcium levels at the expense of increased bone resorption. Laboratory findings in both neonates of this report resembled this situation (**Figure 1**) The demand for calcium may result in pre – and perinatal fractures, as seen in patient 2 (**Figure 2**) (16, 20). However, paternal inheritance does not always lead to NHPT but may only cause FHH, as shown in the first brother of this report (25). In contrast, in pregnancies affected by maternally derived FHH, mother and fetus share the need for increased calcium levels and stimulation of fetal PTH is not required for calcium homeostasis (26).

In fact, the majority of the reported neonatal FHH cases are caused by de-novo or paternally derived mutations in *CaSR*, supporting the notion that maternally inherited FHH usually remains asymptomatic (11, 23, 27). Like patient 2 of this report Tonyushkina et al. reported the case of a female infant with paternally derived FHH (*CaSR* (c.1664T>C) het) who was affected by multiple fractures and bilateral femoral bowing *in utero* (28) and like the older child of this report, prematurely born infants with paternally inherited CaSR mutations were reported by Harris et al. and Fox et al. While the reason for premature delivery in patient 1 of this report was rupture of membranes (25 weeks), acute antepartum hemorrhage (27 weeks) and progressive preeclampsia (34 weeks) were reported in the other two cases (20, 29).

A modifying factor in the phenotypic variability of heterozygous *CaSR* mutations may be the maternal vitamin D status. Schwarz et al. reported the rare case of an infant with NHPT, based on a maternal heterozygous mutation in *CaSR*. (30) In this case NHPT might have risen based on insufficient calcium supply of the fetus, e.g. *via* severe maternal vitamin D deficiency (11). Consistent, Zajickova et al. presented the case of a newborn who inherited a heterozygous mutation from her father and presented with a milder phenotype. When studying the modifying effect of exogenous factors, the authors demonstrated an effect of adequate vitamin D levels in the infant, born to a healthy, yet vitamin D insufficient mother. This patient with low 25OHD level profited from an early start of vitamin D supplementation (21)

A possible reason for the different clinical presentation of the two patients of this report might be the gestational week at birth. Patient 2 was born full-term with a more severe disease and multiple fractures, which at least partly were probably present in utero. Due to the longer duration of the pregnancy, this child was longer exposed to the relatively hypocalcemic intra-uterine environment. In contrast, patient 1 was born at 25 + 6 weeks gestation and received calcium supplementation allowing for better mineralization during the third trimester. In utero, 80% of mineral is absorbed during the third trimester, resulting in an increase of trans-placenta calcium uptake from 60 mg/day of calcium at week 24 of gestation to between 300 and 350 mg/day during the last 6 weeks of the pregnancy (31, 32). Another potential mechanism for the different clinical presentation of the two patients of this report might be maternal vitamin D status during Gestation, as maternal vitamin D supply und calcium status plays a role for the clinical outcome of neonates with CaSR mutations (21).

These findings and the knowledge on the pathophysiology of FHH should have practical implications for the management of pregnancies affected by FHH and genetic counseling of patients with FHH. This becomes particularly relevant in light of the recent publication by Dershem et al. who presented results from whole exome analyses in 51,289 probands of the DiscovEHR cohort in northern USA and detected a prevalence of *CaSR* mutations of 74.1 per 100.000. Of those, 49/100.000 show a clinical FHH1 phenotype. The authors state that these results indicate a prevalence of FHH comparable to that of primary hyperparathyroidism (PHPT). If these findings can be replicated, FHH cannot be considered a rare disease anymore (33, 34). Assuming a prevalence of 74/100.000, about 580 neonates would have been affected by inherited FHH (with about 50% paternal inheritance) and an unknown number of *de novo* cases, based on the 780.000 livebirths in Germany in 2019. These numbers are difficult to interpret, given the fact that NHPT is reported so rarely in the literature.

Given the range of possible neonatal outcomes, Ghaznavi et al., recommend offering genetic counseling to all pregnant women with confirmed FHH or a partner with FHH (10). This seems reasonable, especially in the light of the data by Dershem et al. (33). Of note, no specific treatment in utero is reported or seems feasible, except for ensuring a sufficient vitamin D and calcium status in the mother.

Treatment of neonatal FHH and NHPT is not always necessary. Spontaneous clinical improvements in infants with NHPT have been reported (35). In fact, spontaneous recovery of neonates should be expected in cases of NHPT based on paternal inheritance, since the counter-regulating maternal environment is no longer influencing the fetal organism, and the neonate is allowed to achieve higher calcium levels and correct the osteopenia, if enough calcium is provided (20)

In very severe cases with clinical signs and fractures treatment with cinacalcet can be considered as in patient 2 of this report. Cinacalcet binds within the transmembrane domain and enhances CaSR sensitivity to extracellular calcium, resulting in normalization of PTH **(** **Figure 1A** **)**. It is not approved for clinical use in pediatrics or FHH (36) but successful off label use in FHH has been reported previously (37–39). Based on the severe phenotype with multiple fractures, it was decided to use cinacalcet off label and discontinue the calcium treatment. The mutation in the family of this report is known to result in defective receptor signaling (13, 22, 23), while the ligand binding is unaffected allowing for potential treatment with calcimimetic drugs (12, 24). However, it remains unclear, whether the use of cinacalcet was necessary or whether a spontaneous improvement over time would have occurred, as reported by others (35).

## Conclusion

Neonates with FHH based on paternally inherited *CaSR* mutations may present with or develop symptomatic hyperparathyroidism and fractures. This clinically relevant neonatal hyperparathyroidism may occur more frequently than currently established. Patients with FHH should be informed about this risk and careful monitoring of the pregnancy and the neonates is necessary.

## Data Availability Statement

The original contributions presented in the study are included in the article/supplementary material. Further inquiries can be directed to the corresponding author.

## Ethics Statement

Ethical review and approval was not required for the study on human participants in accordance with the local legislation and institutional requirements. Written informed consent to participate in this study was provided by the participants’ legal guardian/next of kin. Written informed consent was obtained from the minor(s)’ legal guardian/next of kin for the publication of any potentially identifiable images or data included in this article.

## Author Contributions

JH, WH, and CG conceptualized and designed the study, drafted the initial manuscript, and reviewed and revised the manuscript. SL, CR, AW-P, and DW collected data, carried out the initial analyses, and reviewed and revised the manuscript. All authors contributed to the article and approved the submitted version.

## Funding

The authors acknowledge support by the Open Access Publication Funds of the Ruhr-University Bochum.

## Conflict of Interest

The authors declare that the research was conducted in the absence of any commercial or financial relationships that could be construed as a potential conflict of interest.

## Publisher’s Note

All claims expressed in this article are solely those of the authors and do not necessarily represent those of their affiliated organizations, or those of the publisher, the editors and the reviewers. Any product that may be evaluated in this article, or claim that may be made by its manufacturer, is not guaranteed or endorsed by the publisher.

## Abbreviations

NHPT, neonatal hyperparathyroidism; CaSR, calcium-sensing receptor; NSHPT, neonatal severe hyperparathyroidism; FHH, familial hypocalciuric hypercalcemia; PTH, parathyroid hormone; TSAP, total serum alkaline phosphatase; PHPT, primary hyperparathyroidism.

## References

[1] Hannan FM Kallay E Chang W Brandi ML Thakker RV. The Calcium-Sensing Receptor in Physiology and in Calcitropic and Noncalcitropic Diseases. Nat Rev Endocrinol (2018) 15(1):33–51. doi: 10.1038/s41574-018-0115-0 30443043 PMC6535143

[2] Lee JY Shoback DM. Familial Hypocalciuric Hypercalcemia and Related Disorders. Best Pract Res: Clin Endocrinol Metab (2018) 32(5):609–19. doi: 10.1016/j.beem.2018.05.004 PMC6767927 30449544

[3] Vannucci L Brandi ML. Familial Hypocalciuric Hypercalcemia and Neonatal Severe Hyperparathyroidism. Front Horm Res (2018) 51:52–62. doi: 10.1159/000491038 30641521

[4] Nesbit MA Hannan FM Howles SA Reed AAC Cranston T Thakker CE. Mutations in AP2S1 Cause Familial Hypocalciuric Hypercalcemia Type 3. Nat Genet (2013) 45(1):93–7. doi: 10.1038/ng.2492 PMC3605788 23222959

[5] Gorvin CM Hannan FM Cranston T Valta H Makitie O Schalin-Jantti C. Cinacalcet Rectifies Hypercalcemia in a Patient With Familial Hypocalciuric Hypercalcemia Type 2 (FHH2) Caused by a Germline Loss-Of-Function Gα11 Mutation. J Bone Miner Res (2018) 33(1):32–41. doi: 10.1002/jbmr.3241 28833550 PMC5813271

[6] Hannan FM Babinsky VN Thakker RV. Disorders of the Calcium-Sensing Receptor and Partner Proteins: Insights Into the Molecular Basis of Calcium Homeostasis. J Mol Endocrinol (2016) 57(3):R127–42. doi: 10.1530/JME-16-0124 PMC5064759 27647839

[7] Brown EM MacLeod RJ. Extracellular Calcium Sensing and Extracellular Calcium. Physiol Rev (2001) 81(1):239–97. doi: 10.1152/physrev.2001.81.1.239 11152759

[8] Christensen SE Nissen PH Vestergaard P Mosekilde L. Familial Hypocalciuric Hypercalcaemia: A Review. Curr Opin Endocrinol Diabetes Obes (2011) 18(6):359–70. doi: 10.1097/MED.0b013e32834c3c7c 21986511

[9] Jones AR Hare MJ Brown J Yang J Meyer C Milat F. Familial Hypocalciuric Hypercalcemia in Pregnancy: Diagnostic Pitfalls. JBMR Plus (2020) 4(6):1–5. doi: 10.1002/jbm4.10362 PMC7285754 32537548

[10] Ghaznavi SA Saad NMA Donovan LE. The Biochemical Profile of Familial Hypocalciuric Hypercalcemia and Primary Hyperparathyroidism During Pregnancy and Lactation: Two Case Reports and Review of the Literature. Case Rep Endocrinol (2016) 2016:1–6. doi: 10.1155/2016/2725486 PMC5120212 27957351

[11] Marx SJ Sinaii N. Neonatal Severe Hyperparathyroidism: Novel Insights From Calcium, PTH, and the CASR Gene. J Clin Endocrinol Metab (2019) 105(4):1061–78. doi: 10.1210/clinem/dgz233 PMC7111126 31778168

[12] Gulcan-Kersin S Kirkgoz T Eltan M Rzayev T Ata P Bilgen H. Cinacalcet as a First-Line Treatment in Neonatal Severe Hyperparathyroidism Secondary to Calcium Sensing Receptor (CaSR) Mutation. Horm Res Paediatr (2020) 34899(41):313–21. doi: 10.1159/000510623 33147586

[13] Reh CMS Hendy GN Cole DEC Jeandron DD. Neonatal Hyperparathyroidism With a Heterozygous Calcium-Sensing Receptor (CASR) R185Q Mutation: Clinical Benefit From Cinacalcet. J Clin Endocrinol Metab (2011) 96(4):707–12. doi: 10.1210/jc.2010-1306 21289269

[14] Marx SJ Attie MF Spiegel AM Levine MA Lasker RD Fox M. An Association Between Neonatal Severe Primary Hyperparathyreoidism and Familial Hypocalciuric Hypercalcemia in Three Kindreds. N Engl J Med (1982) 306(5):257–64. doi: 10.1056/NEJM198202043060502 7054696

[15] Brown EM. Clinical Lessons From the Calcium-Sensing Receptor. Nat Clin Pract Endocrinol Metab (2007) 3(2):122–33. doi: 10.1038/ncpendmet0388 17237839

[16] Obermannova B Banghova K Sumník Z Dvorakova HM Betka J Fencl F. Unusually Severe Phenotype of Neonatal Primary Hyperparathyroidism Due to a Heterozygous Inactivating Mutation in the CASR Gene. Eur J Pediatr (2009) 168(5):569–73. doi: 10.1007/s00431-008-0794-y 18751724

[17] Pollak MR Brown EM Chou YHW Hebert SC Marx SJ Stelnmann B. Mutations in the Human Ca2+-Sensing Receptor Gene Cause Familial Hypocalciuric Hypercalcemia and Neonatal Severe Hyperparathyroidism. Cell (1993) 75(7):1297–303. doi: 10.1016/0092-8674(93)90617-Y 7916660

[18] Pearce SHS Trump D Wooding C Besser GM Chew SL Grant DB. Calcium-Sensing Receptor Mutations in Familial Benign Hypercalcemia and Neonatal Hyperparathyroidism. J Clin Invest (1995) 96(6):2683–92. doi: 10.1172/JCI118335 PMC185975 8675635

[19] Heath H Odelberg S Jackson CE Teh BT Hayward N Larsson C. Clustered Inactivating Mutations and Benign Polymorphisms of the Calcium Receptor Gene in Familial Benign Hypocalciuric Hypercalcemia Suggest Receptor Functional Domains. J Clin Endocrinol Metab (1996) 81(4):1312–7. doi: 10.1210/jcem.81.4.8636323 8636323

[20] Fox L Sadowsky J Pringle KP Kidd A Murdoch J Cole DEC. Neonatal Hyperparathyroidism and Pamidronate Therapy in an Extremely Premature Infant. Pediatrics (2007) 120(5):e1350–4. doi: 10.1542/peds.2006-3209 17974727

[21] Zajickova K Vrbikova J Canaff L Pawelek PD Goltzman D Hendy GN. Identification and Functional Characterization of a Novel Mutation in the Calcium-Sensing Receptor Gene in Familial Hypocalciuric Hypercalcemia: Modulation of Clinical Severity by Vitamin D Status. J Clin Endocrinol Metab (2007) 92(7):2616–23. doi: 10.1210/jc.2007-0123 17473068

[22] Bai M Quinn S Trivedi S Kifor O Pearce SHS Pollak MR. Expression and Characterization of Inactivating and Activating Mutations in the Human Ca2+(o)-Sensing Receptor. J Biol Chem (1996) 271(32):19537–45. doi: 10.1074/jbc.271.32.19537 8702647

[23] Bai M Pearce SHS Kifor O Trivedi S Stauffer UG Thakker RV. *In Vivo* and *In Vitro* Characterization of Neonatal Hyperparathyroidism Resulting From a De Novo, Heterozygous Mutation in the Ca2+-Sensing Receptor Gene: Normal Maternal Calcium Homeostasis as a Cause of Secondary Hyperparathyroidism in Familial Benign Hyp. J Clin Invest (1997) 99(1):88–96. doi: 10.1172/JCI119137 9011580 PMC507771

[24] Forman TE Niemi AK Prahalad P Shi RZ Nally LM. Cinacalcet Therapy in an Infant With an R185Q Calcium-Sensing Receptor Mutation Causing Hyperparathyroidism: A Case Report and Review of the Literature. J Pediatr Endocrinol Metab (2019) 32(3):305–10. doi: 10.1515/jpem-2018-0307 30730839

[25] Glaudo M Letz S Quinkler M Bogner U Elbelt U Strasburger CJ. Heterozygous Inactivating CaSR Mutations Causing Neonatal Hyperparathyroidism: Function, Inheritance and Phenotype. Eur J Endocrinol (2016) 175(5):421–31. doi: 10.1530/EJE-16-0223 27666534

[26] Murthy A Murthy NPN Ashawesh K Kulambil Padinjakara RN Anwar A. Familial Hypocalciuric Hypercalcaemia and Pregnancy Outcome. Endocr Abstr (2009) 19:P16.

[27] Waller S Kurzawinski T Spitz L Thakker R Cranston T Pearce S. Neonatal Severe Hyperparathyroidism: Genotype/phenotype Correlation and the Use of Pamidronate as Rescue Therapy. Eur J Pediatr (2004) 163(10):589–94. doi: 10.1007/s00431-004-1491-0 15241688

[28] Tonyushkina KN O’Connor S Dunbar NS. A Novel CaSR Mutation Presenting as a Severe Case of Neonatal Familial Hypocalciuric Hypercalcemia. Int J Pediatr Endocrinol (2012) 2012(1):1–7. doi: 10.1186/1687-9856-2012-13 22620673 PMC3465174

[29] Harris SS Joseph A. Neonatal Hyperparathyroidism: The Natural Course in the Absence of Surgical Intervention. Pediatrics (1989) 83(1):53–6. 2909976

[30] Schwarz P Larsen NE Lønborg Friis IM Lillquist K Brown EM Gammeltoft S. Familial Hypocalciuric Hypercalcemia and Neonatal Severe Hyperparathyroidism Associated With Mutations in the Human Ca2+-Sensing Receptor Gene in Three Danish Families. Scand J Clin Lab Invest (2000) 60(3):221–8. doi: 10.1080/003655100750044875 10885494

[31] Ryan BA Kovacs CS. Maternal and Fetal Vitamin D and Their Roles in Mineral Homeostasis and Fetal Bone Development. J Endocrinol Invest (2021) 44(4):643–59. doi: 10.1007/s40618-020-01387-2 32772256

[32] Rauch F Schoenau E. Skeletal Development in Premature Infants: A Review of Bone Physiology Beyond Nutritional Aspects. Arch Dis Child: Fetal Neonatal Ed (2002) 86(2):82–6. doi: 10.1136/fn.86.2.F82 PMC1721373 11882548

[33] Dershem R Gorvin CM Metpally RPR Krishnamurthy S Smelser DT Hannan FM. Familial Hypocalciuric Hypercalcemia Type 1 and Autosomal-Dominant Hypocalcemia Type 1: Prevalence in a Large Healthcare Population. Am J Hum Genet (2020) 106(6):734–47. doi: 10.1016/j.ajhg.2020.04.006 PMC7273533 32386559

[34] Yeh MW Ituarte PHG Zhou HC Nishimoto S Liu I-LA Harari A. Incidence and Prevalence of Primary Hyperparathyroidism in a Racially Mixed Population. J Clin Endocrinol Metab (2013) 98(3):1122–9. doi: 10.1210/jc.2012-4022 PMC3590475 23418315

[35] Wilkinson H James J. Self Limiting Neonatal Primary Hyperparathyroidism Associated With Familial Hypocalciuric Hypercalcaemia. Arch Dis Child (1993) 69(3 SPEC NO):319–21. doi: 10.1136/adc.69.3_Spec_No.319 PMC1029502 8215575

[36] Wüthrich RP Martin D Bilezikian JP. The Role of Calcimimetics in the Treatment of Hyperparathyroidism. Eur J Clin Invest (2007) 37(12):915–22. doi: 10.1111/j.1365-2362.2007.01874.x 18036025

[37] Timmers HJLM Karperien M Hamdy NAT De Boer H Hermus ARMM. Normalization of Serum Calcium by Cinacalcet in a Patient With Hypercalcaemia Due to a De Novo Inactivating Mutation of the Calcium-Sensing Receptor. J Internal Med (2006) 260(2):177–82. doi: 10.1111/j.1365-2796.2006.01684.x 16882283

[38] Festen-Spanjer B Haring CM Koster JB Mudde AH. Correction of Hypercalcaemia by Cinacalcet in Familial Hypocalciuric Hypercalcaemia. Clin Endocrinol (2007) 68:324–5. doi: 10.1111/j.1365-2265.2007.03027.x 17803689

[39] Mayr B Schnabel D Do Rr HGN Schöfl C. Gain and Loss of Function Mutations of the Calcium-Sensing Receptor and Associated Proteins: Current Treatment Concepts. Eur J Endocrinol (2016) 174(5):R189–208. doi: 10.1530/EJE-15-1028 26646938

